# Influence of the supramolecular architecture on the magnetic properties of a Dy^III^ single-molecule magnet: an ab initio investigation

**DOI:** 10.3762/bjnano.5.236

**Published:** 2014-11-27

**Authors:** Julie Jung, Olivier Cador, Kevin Bernot, Fabrice Pointillart, Javier Luzon, Boris Le Guennic

**Affiliations:** 1 Institut des Sciences Chimiques de Rennes, UMR 6226 CNRS - Université de Rennes 1, 263 Avenue du Général Leclerc, 35042 Rennes Cedex, France; 2 INSA, ISCR, UMR 6226, Université Européenne de Bretagne, 35708 Rennes, France; 3 Instituto de Ciencia de Materiales de Aragon, CSIC–Universidad de Zaragoza, Pedro Cerbuna 12, 50009 Zaragoza, Spain; 4 Centro Universitario de la Defensa, Academia General Militar, Zaragoza, Spain

**Keywords:** ab initio calculations, dysprosium, magnetic properties, single-molecule magnets, supramolecular effects

## Abstract

Single-crystal angular-resolved magnetometry and wavefunction-based calculations have been used to reconsider the magnetic properties of a recently reported Dy^III^-based single-molecule magnet, namely [Dy(hfac)_3_(L^1^)] with hfac^−^ = 1,1,1,5,5,5-hexafluoroacetylacetonate and L^1^ = 2-(4,5-bis(propylthio)-1,3-dithiol-2-ylidene)-6-(pyridin-2-yl)-5H-[1,3]dithiolo[4',5':4,5]benzo[1,2-d]imidazole. The magnetic susceptibility and magnetization at low temperature are found to be strongly influenced by supramolecular interactions. Moreover, taking into account the hydrogen-bond networks in the calculations allows to explain the orientation of the magnetic axes. This strongly suggests that hydrogen bonds play an important role in the modulation of the electrostatic environment around the Dy^III^ center that governs the nature of its magnetic ground-state and the orientation of its anisotropy axes. We thus show here that SMM properties that rely on supramolecular organization may not be transferable into single-molecule devices.

## Introduction

At the molecular level, single-molecule magnets (SMMs) can be seen as magnets in which the magnetic information relies on the magnetic moment of the molecule and its magnetic anisotropy [1]. Most of SMMs have been characterized as bulk crystalline material in which intermolecular magnetic interactions are expected to be negligible when compared to the intramolecular ones. The magnetic properties of a compound have then a molecular origin. However the “single-molecule” terminology can be misleading. In fact, in some particular cases, supramolecular interactions have been evidenced to play a significant role in SMM behavior. For instance, in Mn aggregates, supramolecular organization generates exchange-biased quantum tunneling [2]. The easiest way to evidence these supramolecular effects is to design a diamagnetic solid solution in which the sample is present at a doping level [3–12]. The investigation of such sample shows drastic differences from the bulk and highlights that a “single-molecule” when embedded in its crystalline matrix does not behave as an isolated object. This sensitivity of SMM to their environment makes their insertion into devices [13–15] trickier than expected. If SMM are considered for quantum information processing [16–19], supramolecular interactions are expected to generate decoherence [20]. If spin-based devices [13] are considered, the influence of supramolecular interactions has to be characterized very well before deposition of the molecule on a surface. This implies new strategies and new investigation tools [21–22]. When the molecule benefits from a well-known architecture [23–24] that can be optimized for grafting [25–26] the magnetic properties of the molecular object can be kept at the surface [27–28]. This is a tremendous breakthrough in magnetic molecular science that opens the way to molecular surface magnetometry [29]. However, in a “core-shell” picture, where the core is the magnetic ion and the shell its organic surrounding, shell deformation upon grafting can drastically impact the properties of the molecule. A good example is Tb-phthalocyanine molecule, which is one of the most efficient SMM [30]. Depending on the surface and the grafting or deposition mode [25,31–33], it can show erratic hysteresis and even some depth- dependence of the magnetic behavior when multilayers are considered [34]. In order to overcome these drawbacks and to understand their origin, many studies have been undertaken on single-crystals to extensively characterize the magnetic anisotropy of the molecules [9–10,35–38] and its evolution with ligand modifications [39–41]. These studies have been performed mainly on lanthanide-based SMMs as these ions are expected to be extremely sensitive to modifications of the surrounding [42–43]. The first strong experimental evidence has been given by the investigation of DyDOTA (where H_4_DOTA = 1,4,7,10-tetraazacyclododecane *N*,*N*′,*N*′′,*N*′′′-tetraacetic acid) the Dy derivative of the famous GdDOTA that is a commercial contrast agent used in MRI [44]. In this molecule, lanthanide coordination is ensured by one DOTA ligand and one water molecule which provides the “contrast properties” of the compound [45]. A general assumption was that these properties were governed by the Ln–O bond that was supposed to be close to the easy magnetization axis of the molecule. Synergistic investigation by single-crystal magnetometry, low temperature luminescence, and wavefunction-based ab initio calculations, has demonstrated that subtle modification of the Dy^III^ environment such as the rotation of the water molecule is enough to be the driving force of the easy-axis orientation in such a molecule [40]. Subsequent investigations have shown that all lanthanides from Tb to Yb are affected in the same way [36]. This reveals that this subtle effect can be considered as a general property of 4f open-shell ions whatever their ground-state parity. This opens the way to close theoretical examinations of Ln-based SMMs as simple electrostatic approaches were not able to reproduce such results [46].

The influence of the surrounding on Ln-based SMM can also be highlighted through a supramolecular point of view. As an example, the special packing of two analogous Yb-based molecules in which H-bonds are present or not, drastically influences the orientation of the magnetic easy axis [12,39]. In the latter, multiconfigurational post-Hartree–Fock calculations demonstrated that the relative position of one hydrogen atom along the N–H^…^O bonding mode tailors its orientation.

In the present article, a Dy^III^-based SMM in which supramolecular effects impact the magnetic properties is investigated on the basis of single-crystal angular-resolved magnetometry and ab initio calculations.

## Results and Discussion

**Figure 1:**
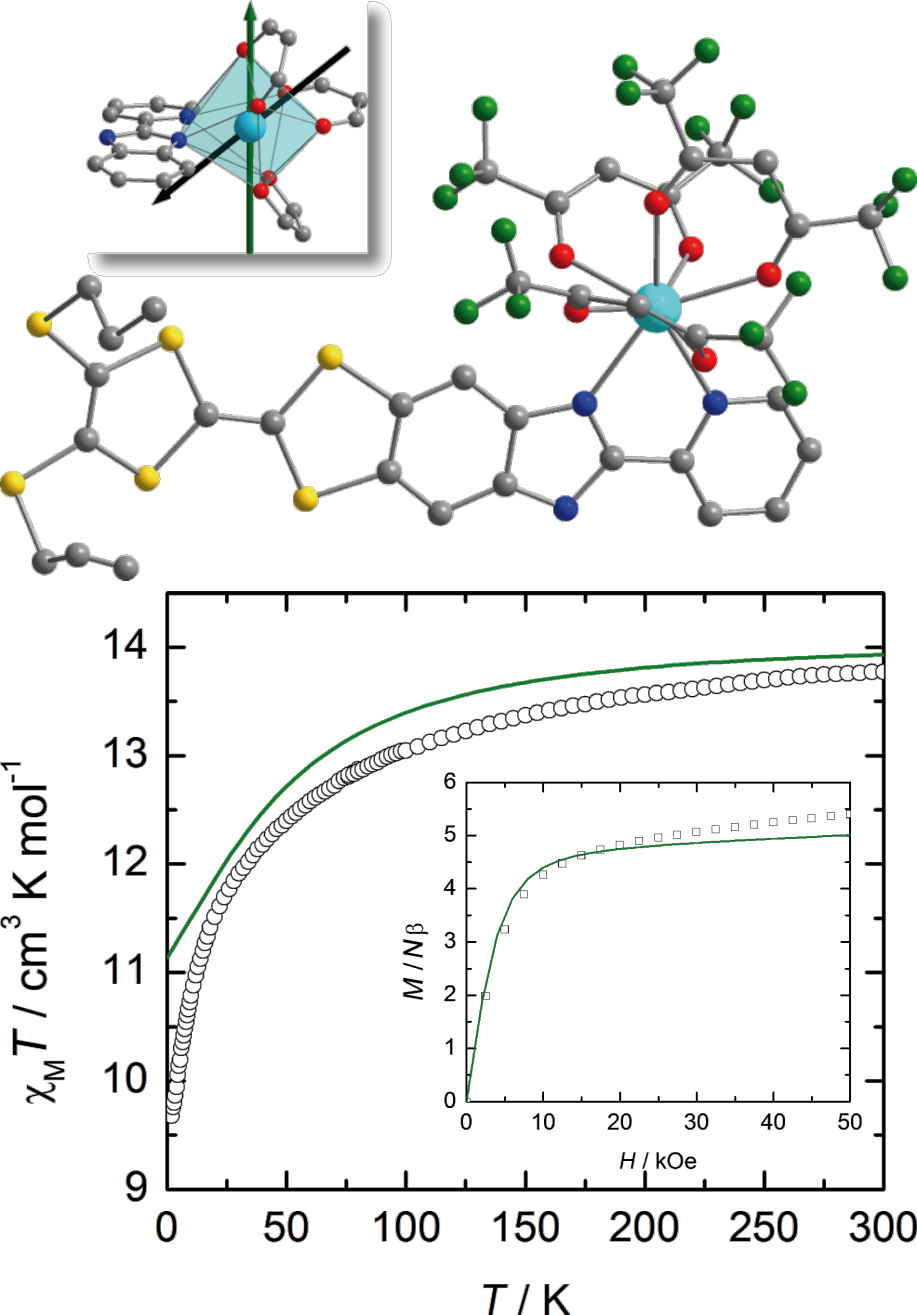
Molecular structure of **Dy1** (top). Dy, O, N, C, S and F atoms are depicted in light blue, red, blue, grey, yellow and green, respectively. H atoms are omitted for clarity. Inset: Experimental (black) and theoretical (green) ground state anisotropy axes are shown on the coordination polyhedron. Thermal variation of *χ*_M_*T* of a solid-state sample of **Dy1** (black circles) with the curve (in green) calculated on the basis of SA-CASSCF/RASSI-SO data (bottom). Inset: field variation of the magnetization at 2 K (black squares) with the computed curve (in green) obtained at the same level of calculation.

We have focused our investigation on two Dy^III^-based complexes that were reported by some of us recently [12]. As a short reminder, both complexes are mononuclear species of the general formula [Dy(hfac)_3_(L^1^)] (**Dy1**) and [Dy(hfac)_3_(L^2^)] (**Dy2**). **Dy1** crystallizes in the triclinic *P*–1 (No. 2) space group with a unit cell composed of mononuclear complexes of the formula [Dy(hfac)_3_(L^1^)] with hfac^−^ = 1,1,1,5,5,5-hexafluoroacetylacetonate and L^1^ = 2-(4,5-bis(propylthio)-1,3-dithiol-2-ylidene)-6-(pyridin-2-yl)-5H-[1,3]dithiolo[4',5':4,5]benzo[1,2-d]imidazole. In this complex, the Dy^III^ ion is surrounded by six oxygen atoms and two nitrogen atoms belonging to three hfac^−^ ligands and one bis-chelating L^1^ ligand (Figure 1). The average Dy–O distances are shorter (2.35(3) Å) than the average Dy–N distances (2.50(6) Å). **Dy2** crystallizes in the monoclinic *P*2_1_/*c* (No. 14) space group and the unit cell is composed of mononuclear complexes of the formula [Dy(hfac)_3_(L^2^)] with L^2^ = 2-(4,5-bis(propylthio)-1,3-dithiol-2-ylidene)-6-(pyridin-2-yl)-5-(pyridin-2-ylmethyl)-5H-[1,3]dithiolo[4',5':4,5]benzo[1,2-d]imidazole (Figure S1, Supporting Information File 1). As for **Dy1**, the Dy^III^ ion is surrounded by six oxygen atoms and two nitrogen atoms belonging to three hfac^−^ ligands and one bis-chelating L^2^ ligand. The average Dy–O and Dy–N distances are equal to 2.34(4) Å and 2.49(5) Å, respectively. The formation of “head to tail” dimers is observed in both compounds.

Despite their identical coordination spheres the experimental magnetic properties of the two compounds differ significantly. Indeed, in the condensed phase the thermal variations of *χ*_M_*T* as well as the field variations of the magnetization at 2 K do not match (Figure 1 and Figure S1, Supporting Information File 1). While for both complexes the high temperature values of *χ*_M_*T* coincide and are close to the expected value for a ^6^H_15/2_ multiplet (14.17 cm^3^·K·mol^−1^) [47], on cooling the values of *χ*_M_*T* of **Dy1** is far below the ones of **Dy2**. On the other hand, the magnetization at 2 K increases linearly for **Dy1** at fields higher than 1 T while it saturates for **Dy2**. The consequences of these differences is that **Dy2** behaves as a SMM in the solid state while **Dy1** does not [12]. However, the latter behaves as a SMM in CH_2_Cl_2_ solution. This drastic difference of behavior between solid state and solution was attributed, with no clear experimental evidence, to the breaking of the hydrogen-bond network in solution. This is what we would like to clarify in the present work.

**Figure 2:**
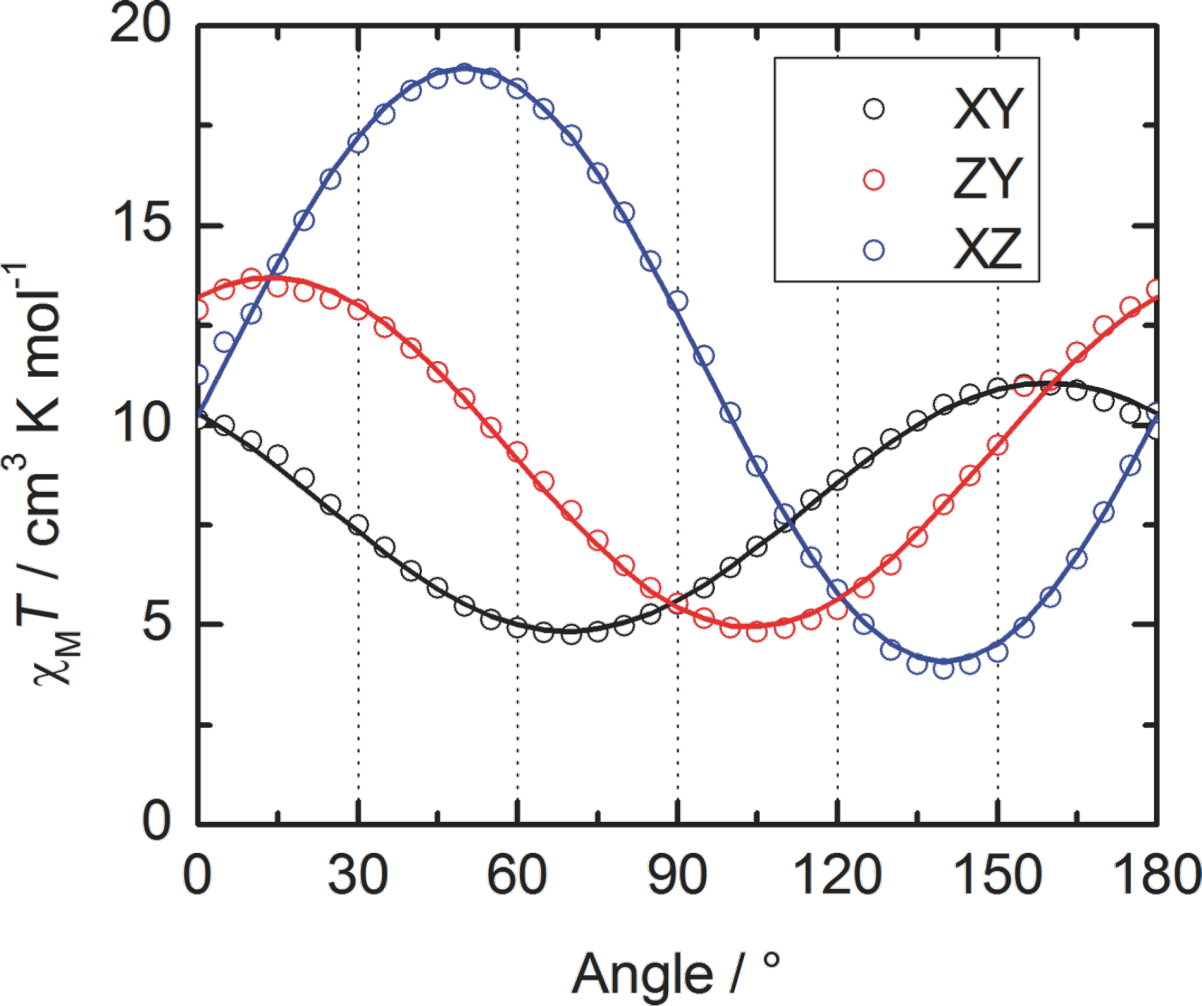
Angular dependence of *χ*_M_*T* measured for **Dy1** in the three orthogonal planes with the best fitted curves as solid lines.

Following this first investigation [12], we took advantage of the uniqueness of the molecule in the *P*–1 space group to perform single-crystal angular-resolved magnetometry for **Dy1** (see Experimental section) as already done in the case of the Yb^III^ derivative [39]. After indexation of the crystal faces through single-crystal diffraction (Figure S2, Supporting Information File 1), the angular dependence of the magnetization was measured in three orthogonal planes (XY, YZ and XZ) at 2 K with an applied magnetic field of 1 kOe (Figure 2). The data were then fitted assuming that *M* = *χ*_M_*H*. Rotation of *H* in the αβ-plane changes the expression of the magnetization to *M*/*H* = *χ*_αα_(cosθ)^2^ + *χ*_ββ_(sinθ)^2^ + 2*χ*_αβ_(sinθ cosθ), where α and β are the directions of the vectors X, Y and Z in a cyclic permutation and θ is the angle between *H* and α (Figure 2). The principal values of the Zeeman tensor in the 1/2 effective spin approximation (*g_z_* = 14.22, *g_y_* = 3.96 and *g_x_* = 9.43) as well as its orientation are extracted (see Supporting Information File 1). First of all, the principal values do not fit with an Ising-type anisotropy (*g_z_* = 20, *g_y_* = *g_x_* = 0) which agrees with the non-SMM behavior of this compound in the solid state. Secondly, the tensor orientation of the ground state is not lying in any special direction (Figure 1).

**Figure 3:**
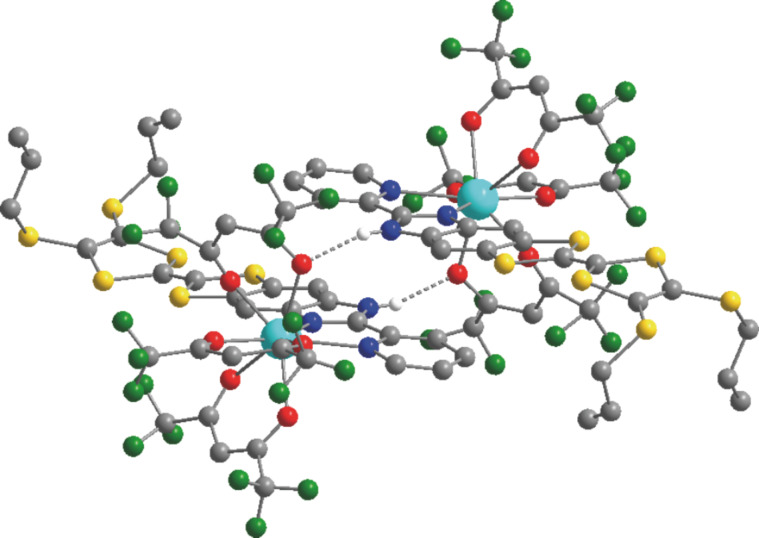
Representation of supramolecular interactions in **Dy1**. Dy, O, N, C, S and F atoms are depicted in light blue, red, blue, grey, yellow and green, respectively. H atoms (except the H atoms involved in hydrogen bonds) are omitted for clarity.

Relativistic ab initio calculations (SA-CASSCF/RASSI-SO) have been performed in order to rationalize the observed magnetic properties of both compounds **Dy1** and **Dy2**. We first attempted to reproduce the magnetic data in solely considering isolated molecules (see Experimental section). For **Dy2** the computed *χ*_M_*T* vs *T* and *M* vs *H* curves almost perfectly match the experimental ones (Figure S1, Supporting Information File 1). On the contrary, this “molecular” approach dramatically fails in the case of **Dy1** with a significant discrepancy between calculated and experimental values at the low temperature limit for *χ*_M_*T* (computed: *χ*_M_*T* = 11.135 cm^3^·K·mol^−1^; experimental: *χ*_M_*T* = 9.67 cm^3^·K·mol^−1^, Figure 1). Also, at 2 K the computed *M* vs *H* curve saturates contrary to the experimental one (Figure 1), a behavior that was already observed for the Yb parents [Yb(hfac)_3_(L^1^)] and [Yb(hfac)_3_(L^2^)] [39]. The disagreement for [Yb(hfac)_3_(L^1^)] was attributed to intermolecular interactions that seem to play a key role in the magnetic properties of this series of complexes. Moreover, the calculated ground state of **Dy1** is almost Ising (see below in Table 1) in contradiction to the solid-state experiments (see above). This result is confirmed by the nature of the calculated ground-state wavefunction that is mainly composed of *M_J_* = 15/2 state with a small contribution of the *M_J_* = 11/2 state. Finally, the orientation of the calculated easy axis differ by more than 57° from the experiment. In short, whereas this “molecular” computational results do not reproduce the solid-state behavior, they are in line with the observations made in solution [12]. The above results showed that a “local” description that only takes into account intramolecular interactions is not able to explain the solid-state magnetism of this complex. As already mentioned in the introduction, subtle geometric effects may change both magnetic susceptibility and orientation of the easy axis [39–40]. Contrary to **Dy2**, intermolecular hydrogen bond networks organize the three dimensional edifice in **Dy1** (Figure 3) [12]. We thus revisit the theoretical interpretation on the basis of these supramolecular interactions.

**Figure 4:**
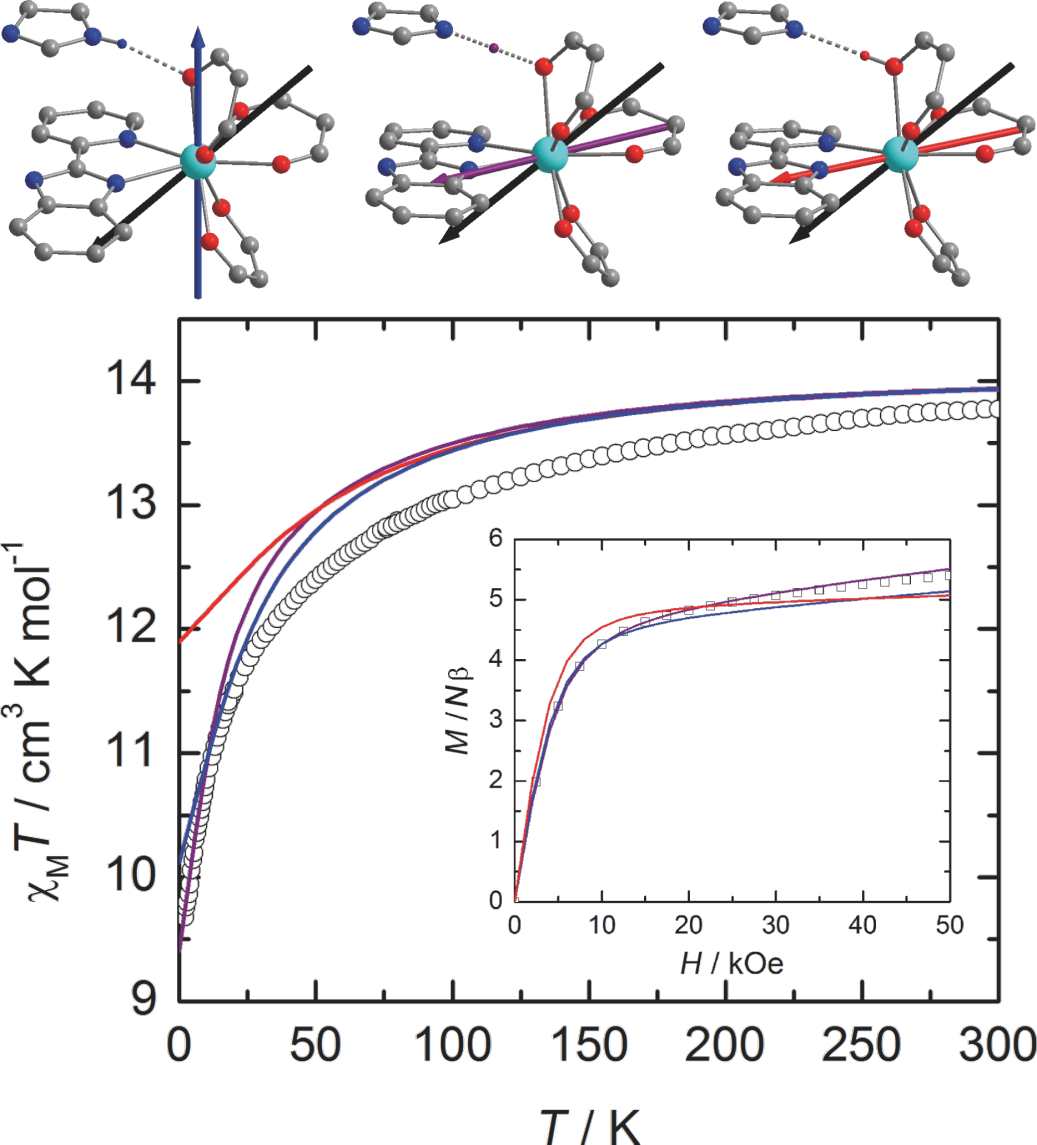
Orientation of the experimental (black) and calculated ground-state anisotropy axes for **Dy1** (top). The orientation of the calculated axis is given for the different positions of the hydrogen atom involved in the hydrogen bond, i.e., from left to right: H_N_ (blue), H_m_ (purple) and H_O_ (red). Thermal variation of *χ*_M_*T* of a solid-state sample of **Dy1** (black circles) with the curve calculated on the basis of SA-CASSCF/RASSI-SO data for the various positions of the H atom (bottom). Inset: field variation of the magnetization at 2 K (black squares) with the computed curve obtained at the same level of calculation.

In **Dy1**, a hydrogen bond is formed between the protonated imidazole ring and the oxygen atom of the neighboring molecule. On the contrary, in **Dy2**, the presence of the 2-methylpyridine arm prevents such weak interactions between neighboring molecules [12]. To mimic this hydrogen bond in the calculations, the neighboring complex in **Dy1** was modeled by an imidazole molecule. Various arbitrary positions of the H atom were considered, i.e., i) at the position calculated from single-crystal X-ray diffraction (H_N_), ii) along the O^…^N axis at a classical O–H distance (H_O_) and iii) equidistant to N and O (H_m_). In order to cover as much as possible of both the long-range interactions and the electronic reorganization that might be induced by this weak interaction, the hydrogen atom involved in the hydrogen bond was described with an extended [3s2p1d] basis set (see Experimental section). First, the presence of this hydrogen bond in the calculations slightly affects the relative energy splitting of the ground-state multiplet. Compared with the non-protonated situation, the whole splitting is slightly reduced for H_m_ and H_N_ whereas it increases for H_O_ (Table 1 and Figure S3, Supporting Information File 1). More importantly, the energy gap between the ground and first excited states is much smaller when the H atom is positioned close to the N atom of the imidazole or in the median position. Thus, the weight of the *M_J_* = ±15/2 state in the ground-state wavefunction is significantly lowered and mixing with other *M_J_* states is observed (Table 1). Concomitantly, the magnetic susceptibility and magnetization curves are progressively closer to the experimental ones (Figure 4). In particular, for the hydrogen atom at the H_m_ position, the low temperature limit for *χ*_M_*T* is well reproduced (computed: *χ*_M_*T* = 9.40 cm^3^·K·mol^−1^; experimental: *χ*_M_*T* = 9.67 cm^3^·K·mol^−1^), as well as the *M* vs *H* curve at 2 K. As shown in Figure 4 the location of the proton has a non-negligible effect on the orientation of the ground state magnetic axis. Whereas this axis is calculated far away from the experimental one if the hydrogen bond is not taken into account (α = 57°) or for H_N_ (α = 67°), the discrepancy is much weaker for H_O_ (α = 27°) and H_m_ (α = 29°, Table 1). As described previously [10,39,41], the orientation of the axis is governed by the variation of the electrostatic potentials generated by the coordinated atoms on the Dy^III^ center (Table S1, Supporting Information File 1). In particular, the charge on the oxygen atom (O5) involved in the hydrogen-bond evolves significantly. This induces large modifications of the charge distribution around Dy^III^ with respect to the position of the hydrogen atom. Based on these observations, it seems thus that H_m_ is the most suited position for this particular H atom. It may signify that at the time scale of the magnetic measurements an “averaged” position of the H atom along the N–H^…^O bond has to be considered.

**Table 1:** Computed ground-state anisotropy tensor for **Dy1** for different positions of the hydrogen atom involved in the hydrogen bond. The weights of the ± *M_J_* components of the calculated ground-state wavefunction, the relative energy of the first excited-state (Δ*E*, cm^−1^) and the angle (*α*, degrees) between the experimental and computed easy axes are also given.

H atom position	*g_x_*	*g_y_*	*g_z_*	± *M_J_* weights of the GS wavefunction	Δ*E*	α

no H	0.08	0.16	18.87	0.85|±15/2>; 0.11|±11/2>; 0.03|±7/2>	91.1	56.9
H_O_	0.02	0.03	19.51	0.94|±15/2>; 0.03|±9/2>; 0.02|±11/2>	109.7	27.1
H_m_	0.83	3.05	17.05	0.77|±15/2>; 0.10|±9/2>; 0.03|±5/2>; 0.03|±1/2>; 0.03|±3/2>	34.1	28.5
H_N_	0.39	1.25	17.94	0.78|±15/2>; 0.12|±11/2>; 0.06|±7/2>; 0.03|±3/2>	48.1	67.0

## Conclusion

The understanding of the subtle mechanisms at the origin of the magnetic properties of molecular materials is a prerequisite before anchoring/grafting these molecular architectures onto surfaces, nanoparticles or graphene-based devices. In this work, we have used wavefunction-based calculations combined with single-crystal angular-resolved magnetometry to reconsider the magnetic properties of a recently proposed Dy^III^-based single-molecule magnet [12]. The magnetic susceptibility and magnetization at low temperature are found to be strongly influenced by supramolecular interactions. Moreover, taking into account the hydrogen-bond networks allows to explain the orientation of the magnetic axes. The computational results suggest that hydrogen bonds have an important influence on the modulation of the electrostatic environment of the Dy^III^ ion. As a consequence it also impacts the nature of the Dy magnetic ground state and the orientation of the magnetic axes. Further investigation of the dynamics of the N–H^…^O bonds and its implication on the magnetic behavior is thus envisaged.

## Experimental

**Computational details.** Ab initio calculations were carried out on model structures of **Dy1** and **Dy2** (see below) by using the SA-CASSCF/RASSI-SO approach, as implemented in the MOLCAS quantum chemistry package (versions 7.6) [48]. In this approach, the relativistic effects are treated in two steps on the basis of the Douglas–Kroll Hamiltonian. First, the scalar terms were included in the basis-set generation and were used to determine the spin-free wavefunctions and energies in the complete active space self consistent field (CASSCF) method [49]. Next, spin-orbit coupling was added within the restricted-active-space-state-interaction (RASSI-SO) method, which uses the spin-free wavefunctions as basis states [50–51]. The resulting wavefunctions and energies are used to compute the magnetic properties and the g-tensors of the lowest states from the energy spectrum by using the pseudo-spin *S* = 1/2 formalism in the SINGLE-ANISO routine [52–53]. The calculated ground state wavefunction were obtained from the RASSI-SO results by using a custom-made program. Cholesky decomposition of the bielectronic integrals was employed to save disk space and speed-up the calculations [54]. For similar reasons, the donor part of the TTF ligand in **Dy1** and **Dy2** was replaced by H atoms [39]. All atoms were represented by ANO-type basis sets from the ANO-RCC library [55–56]. The following contractions were used: [9s8p5d4f3g1h] for the Dy ion, [4s3p2d] for the O and N atoms of the first coordination sphere of the Dy ion, [3s2p] for the C, F and remaining N atoms, [3s2p1d] for the H atom involved in the hydrogen bond and [2s] for all the other H atoms. The active space of the self consistent field (CASSCF) method consisted of the nine 4f electrons of the Dy ion spanning the seven 4f orbitals. State-averaged CASSCF calculations were performed for all of the sextets (21 roots) and quadruplets (224 roots) of the Dy ion. Only 148 quadruplets were added to the 21 sextets to mix through spin–orbit coupling in RASSI-SO. In this case, there was no need to add more quadruplet or doublet roots to converge the wavefunctions and energies of the ground multiplet (^6^H_15/2_) of the Dy ion. The anisotropy tensor, the energy of the eight Kramer doublets of the ground spin–orbit state, as well as the temperature-dependent magnetic susceptibility and the molar magnetization at 2 K were computed to support experimental results. Atomic charges were computed by using the LoProp approach [57].

**Magnetic measurements.** Angular-resolved magnetometry was performed on a single-crystal of **Dy1** with a Quantum Design MPMS-XL SQUID magnetometer by using the horizontal-rotator option. The background of the sample holder was subtracted.

## Supporting Information

Supporting information features molecular structure and magnetic properties of **Dy2**, as well as susceptibility tensor and calculated charges and potentials of **Dy1**.

File 1 Additional experimental data.
