# Supplementary material for: Influence of the supramolecular architecture on the magnetic properties of a DyIII single-molecule magnet: an ab initio investigation
Source: Beilstein J Nanotechnol. 2014 Nov 27;5:2267–74. doi: 10.3762/bjnano.5.236 (PMC4273297; doi:10.3762/bjnano.5.236)
Supplement: File 1 — Additional experimental data. [file Beilstein_J_Nanotechnol-05-2267-s001.pdf]

Supporting Information

for

Influence of the supramolecular architecture on the magnetic properties of a Dy^{III} single-molecule magnet: an ab initio investigation

Julie Jung[1], Olivier Cador[1], Kevin Bernot[2], Fabrice Pointillart[1], Javier Luzon[3,4] and Boris Le Guennic*[1]

Address: [1]Institut des Sciences Chimiques de Rennes, UMR 6226 CNRS - Université de Rennes 1, 263 Avenue du Général Leclerc, 35042 Rennes Cedex, France; [2]INSA, ISCR, UMR 6226, Université Européenne de Bretagne, 35708 Rennes, France; [3]Instituto de Ciencia de Materiales de Aragon, CSIC–Universidad de Zaragoza, Pedro Cerbuna 12, 50009 Zaragoza, Spain and [4]Centro Universitario de la Defensa, Academia General Militar, Zaragoza, Spain

Email: Boris Le Guennic* - boris.leguennic@univ-rennes1.fr

* Corresponding author

Additional experimental data

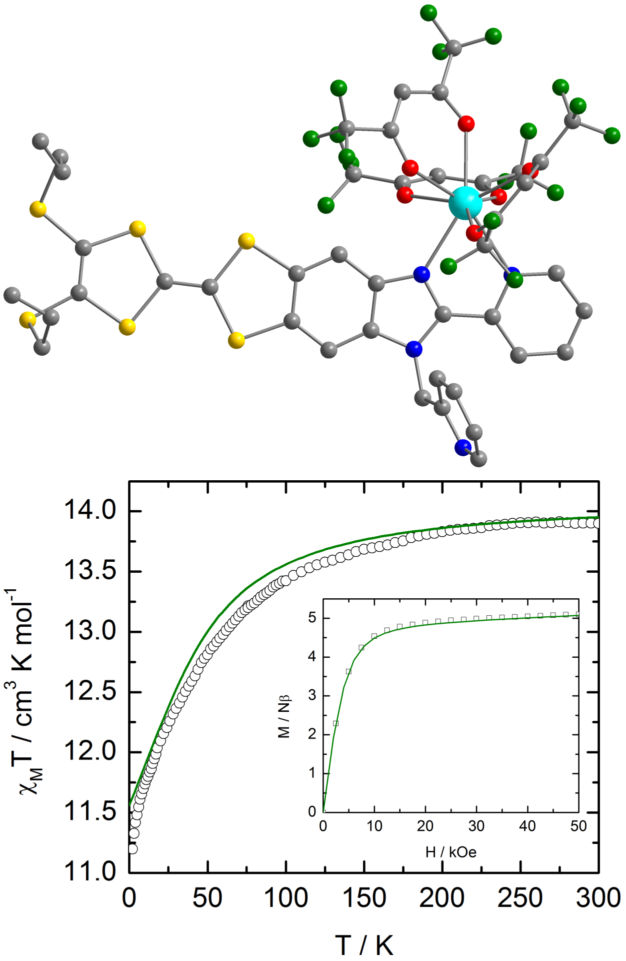

Figure S1: Molecular structure of **Dy2** (top). Dy, O, N, C, S and F atoms are depicted in light blue, red, blue, grey, yellow and green, respectively. H atoms are omitted for clarity. Thermal variation of $\chi_M T$ of a solid-state sample of **Dy2** (black circles) with the curve (in green) computed on the basis of SA-CASSCF/RASSI-SO calculations (bottom). Inset: field variation of the magnetization at 2 K (black squares) with the computed curve (in green) obtained at the same level of calculation.

Susceptibility tensor in the crystal frame (XYZ) for Dy1.

The susceptibility tensor in the crystal reference frame is then equal to:

$$\chi_M T = \begin{pmatrix} 10.136 & -2.222 & 7.313 \\ -2.222 & 5.763 & 2.545 \\ 7.313 & 2.545 & 12.888 \end{pmatrix} \mathrm{cm}^3 \cdot \mathrm{K} \cdot \mathrm{mol}^{-1}$$

Principal values and direction of the susceptibility tensor in the XYZ crystal frame are extracted in diagonalizing the previous matrix:

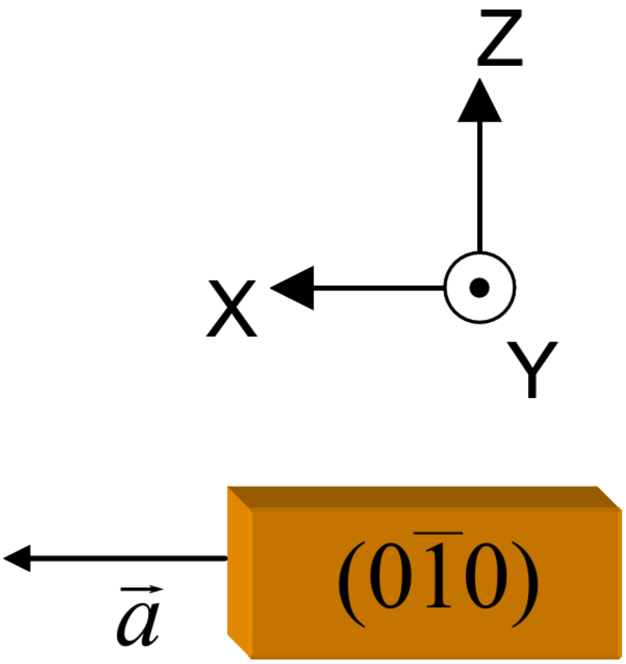

Figure S2: Oriented single-crystal of **Dy1** with the XYZ crystal reference frame.

$$\chi_{xx}T\begin{pmatrix}0.499\\-0.787\\-0.262\end{pmatrix}=8.340,\ \chi_{yy}T\begin{pmatrix}-0.594\\-0.615\\0.518\end{pmatrix}=1.470,\ \chi_{zz}T\begin{pmatrix}0.630\\0.043\\0.775\end{pmatrix}=18.98\ \text{cm}^3\cdot\text{K}\cdot\text{mol}^{-1}$$

In the effective spin 1/2 approximation the principal g values are equal to: g_z = 14.22, g_y = 3.96 and g_x = 9.43.

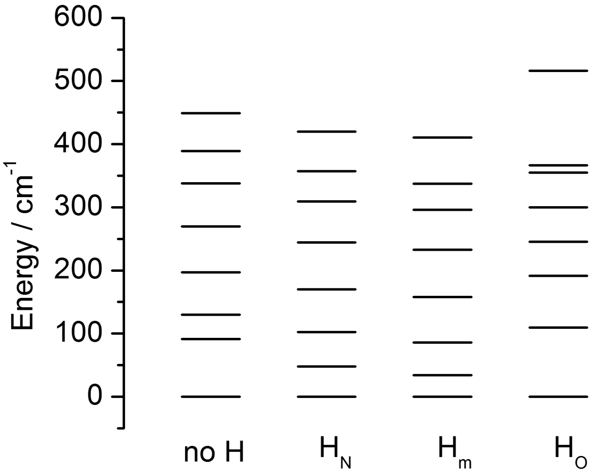

Figure S3: Splitting of the $^6H_{15/2}$ manifold of **Dy1** evaluated from ab initio calculations.

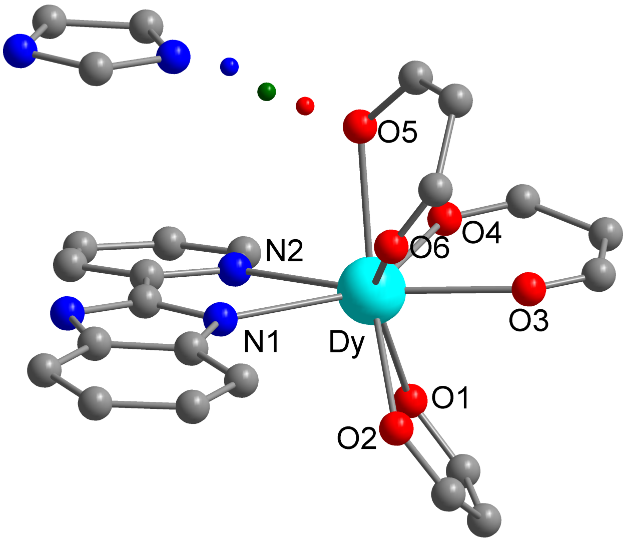

Figure S4: Labels of the atoms in the first coordination sphere of Dy in **Dy1**.

Table S1: Distances between the Dy center and atoms X^i of the first coordination sphere (in Å) for **Dy1**. Labels are given in Figure S4. Calculated charge (q, in e^-) and electrostatic potential (V_E = q/r) for different positions of the hydrogen atom involved in the hydrogen bond. $V_E < 0$ ($V_E > 0$) corresponds to an attractive (repulsive) potential.

		**no H**		$\mathbf{H_N}$		$\mathbf{H_O}$		$\mathbf{H_m}$	
$\mathbf{X^i}$	**Dy-$\mathbf{X^i}$**	**q**	$\mathbf{V_E}$	**q**	$\mathbf{V_E}$	**q**	$\mathbf{V_E}$	**q**	$\mathbf{V_E}$
Dy	—	2.53	—	2.53	—	2.52	—	2.53	—
O1	2.327	−0.73	−0.32	−0.74	−0.32	−0.74	−0.32	−0.74	−0.32
O2	2.319	−0.65	−0.28	−0.65	−0.28	−0.66	−0.28	−0.65	−0.28
O3	2.314	−0.63	−0.27	−0.66	−0.28	−0.67	−0.29	−0.66	−0.29
O4	2.349	−0.64	−0.27	−0.66	−0.28	−0.66	−0.28	−0.66	−0.28
O5	2.388	−0.73	−0.31	−0.76	−0.32	−0.55	−0.23	−0.71	−0.30
O6	2.378	−0.58	−0.25	−0.57	−0.24	−0.50	−0.21	−0.54	−0.23
N1	2.451	−0.45	−0.18	−0.46	−0.19	−0.49	−0.20	−0.47	−0.19
N2	2.538	−0.35	−0.14	−0.36	−0.14	−0.39	−0.15	−0.37	−0.15
